# Body and Soul; or Life, Mind, and Matter, Considered as to Their Peculiar Nature, and Combined Condition in Living Things; with a View to Render the Physiology of Life and Mind More Easily Understood by the General Reader

**Published:** 1847-07

**Authors:** 


ART. X.—*Body and Soul; or Life, Mind, and Matter, considered as to their peculiar nature, and combined condition in living things; with a view to render the Physiology of Life and Mind more easily understood by the general reader.* By GEORGE REDFORD, M.R.C.S. &c.—*London,* 1847, 8vo, pp. 232.

THE title of this work did not excite in us any very favorable prepossession as to its character; since it appeared to indicate one of those attempts to obtain the attention of the public by a sort of clap-trap advertisement, which are so frequently made by half-educated pretenders; or one of those ambitious essays at philosophical composition, which serve to indicate nothing else than the shallowness and self-sufficiency of their authors. After an examination of its contents, however, we feel it right to state that the book cannot be justly referred to either of these categories. It contains a good deal of matter which will be novel and interesting to the general reader, gleaned from the writings of Müller and other physiologists and psychologists; and this is arranged in a manner that will no doubt be attractive to such as have not previously thought deeply on the subjects which the work embraces. Although we cannot give it very high praise, yet, on the other hand, we do not feel called upon to pass a severe judgment upon it. The author enters into his subject in a good spirit; that of endeavouring to convey some definite information upon it to those "who are sometimes mystified or shallowly enlightened by the confident explanations of materialists, over-chemical chemists, phrenologists, and mesmerists." He laments, not unreasonably, that so little can be said in explanation of its mysteries; "still," as he justly remarks,

"It is generally useful to know what there is that we do *not* know, and how far explanation may be carried, especially that we may avoid that too common disposition to throw a veil of mystery, and a sort of almighty incomprehensibility, over subjects which are really perhaps capable of reduction to the effect of general laws, without the possibility of detracting from that reverence which belongs to the mysterious omnipotence of the Deity."

The work consists of three parts: the first treating of *Life*, the second of *Mind*, and the third of the *Combined Phenomena of Life and Mind*. Notwithstanding our disagreement with the author on many points, we regard his treatise as on the whole well adapted to his object, and as likely to do good by the information it imparts and the incitements which it offers to a deeper study of the phenomena of which it treats.

